# A Test of Highly Optimized Tolerance Reveals Fragile Cell-Cycle Mechanisms Are Molecular Targets in Clinical Cancer Trials

**DOI:** 10.1371/journal.pone.0002016

**Published:** 2008-04-23

**Authors:** Satyaprakash Nayak, Saniya Salim, Deyan Luan, Michael Zai, Jeffrey D. Varner

**Affiliations:** 1 Department of Chemical and Biomolecular Engineering, Cornell University, Ithaca, New York, United States of America; 2 Department of Biological and Environmental Engineering, Cornell University, Ithaca, New York, United States of America; IBM Thomas J. Watson Research Center, United States of America

## Abstract

Robustness, a long-recognized property of living systems, allows function in the face of uncertainty while fragility, i.e., extreme sensitivity, can potentially lead to catastrophic failure following seemingly innocuous perturbations. Carlson and Doyle hypothesized that highly-evolved networks, e.g., those involved in cell-cycle regulation, can be resistant to some perturbations while highly sensitive to others. The “robust yet fragile” duality of networks has been termed Highly Optimized Tolerance (HOT) and has been the basis of new lines of inquiry in computational and experimental biology. In this study, we tested the working hypothesis that cell-cycle control architectures obey the HOT paradigm. Three cell-cycle models were analyzed using monte-carlo sensitivity analysis. Overall state sensitivity coefficients, which quantify the robustness or fragility of a given mechanism, were calculated using a monte-carlo strategy with three different numerical techniques along with multiple parameter perturbation strategies to control for possible numerical and sampling artifacts. Approximately 65% of the mechanisms in the G1/S restriction point were responsible for 95% of the sensitivity, conversely, the G2-DNA damage checkpoint showed a much stronger dependence on a few mechanisms; ∼32% or 13 of 40 mechanisms accounted for 95% of the sensitivity. Our analysis predicted that CDC25 and cyclin E mechanisms were strongly implicated in G1/S malfunctions, while fragility in the G2/M checkpoint was predicted to be associated with the regulation of the cyclin B-CDK1 complex. Analysis of a third model containing both G1/S and G2/M checkpoint logic, predicted in addition to mechanisms already mentioned, that translation and programmed proteolysis were also key fragile subsystems. Comparison of the predicted fragile mechanisms with literature and current preclinical and clinical trials suggested a strong correlation between efficacy and fragility. Thus, when taken together, these results support the working hypothesis that cell-cycle control architectures are HOT networks and establish the mathematical estimation and subsequent therapeutic exploitation of fragile mechanisms as a novel strategy for anti-cancer lead generation.

## Introduction

The capability to gather protein-protein and protein-DNA interaction data, for example using the Yeast Two-Hybrid (Y2H) system [1], [2], Fluorescence Resonance Energy Transfer (FRET) techniques [3], quantitative Mass Spectrometry (MS) proteomic or Chromatin Immunoprecipitation (ChIP)-DNA micro-array techniques [4], [5] has far outstripped our ability to understand it. Transforming large-scale interaction data into a better understanding of the biomolecular networks underlying disease progression and eventually to new therapies requires integrative tools and strategies. Perhaps one strategy to leverage our knowledge of interaction networks into efficacious therapies would be to identify and exploit weak or fragile mechanisms while avoiding the manipulation of robust network interactions.

Robustness, a long-recognized property of living systems and networks, allows function in the face of uncertainty while fragility, i.e., extreme sensitivity, can potentially lead to catastrophic failure following seemingly innocuous perturbations [6]–[10]. Different factors can influence why elements of a network are robust or fragile. Venkatasubramanian and co-workers demonstrated that the structure of complex networks can result from a trade-off between efficiency and robustness [11] while You and Yin explored how the environment has shaped the robust properties of bacteriophage T7 [12]. Leibler computationally predicted and later experimentally verified robust features of chemotaxis control networks [13] and Stelling *et al.*, reviewed several examples of robust biological networks [9]. Perhaps no better example of robustness can be found than cell division. The cell-cycle is one of the most fundamental and highly controlled processes in biology. The decision to divide is tightly regulated integrating extracellular signals, such as growth factors and hormones, with intracellular cues that coordinate events leading to division. However, despite extensive control and surveillance subsystems guiding the progression of cells through the division cycle, malfunctions do occur as evidenced by the uncontrolled proliferation underlying many cancers [14]. Thus, while evolutionary pressure may have programmed cells to be robust to shifting nutritional environments or varying growth factor availability, perhaps rare challenges could result in unforeseen consequences. For example, exposure to radiation, exotic chemicals (carcinogens) or even Single Nucleotide Polymorphisms (SNPs) may cause seemingly innocuous changes which manifest themselves in the breakdown of cell-cycle logic. Carlson and Doyle have hypothesized that highly-evolved networks can be resistant to some perturbations while extremely sensitive to others. The “robust yet fragile” duality of networks and systems has been termed Highly Optimized Tolerance (HOT) and has been the basis of new lines of inquiry in computational and experimental biology [10].

Sensitivity analysis is an enabling tool for the investigation of robustness and fragility in networks relevant to human health and more generally for model-based knowledge discovery. Cho *et al.*, used sensitivity analysis to study TNF-α-mediated NF-kβ signaling where parametric uncertainty was addressed using a monte-carlo parameter sampling protocol; a family of random parameter sets, generated from the best parameter guess, was used to calculate the sensitivity profile in a region of parameter space [15]. Bullinger and coworkers explored the robustness of models of programmed cell death or apoptosis [16] while Stelling *et al.*, computationally identified points of robustness and fragility, using monte-carlo sensitivity analysis and Overall State Sensitivity Coefficients (OSSCs), in models of circadian rhythm [17]. Mahdavi *et al.*, employed sensitivity analysis to better understand stem cell differentiation [18], while Luan *et al.*, used an uncertain mechanistic model of the coagulation cascade in combination with monte-carlo sensitivity analysis, to show that computationally derived sensitive mechanisms were consistent with anticoagulation therapeutic strategies [19]. Sensitivity analysis has also been used to integrate model identification and discrimination with optimal experimental design. Several optimal experimental design and model identification studies are resident in the literature [20]–[24] along with many techniques to estimate sensitivity coefficients for models composed of ordinary differential equations, differential algebraic and stochastic equations [25]–[28].

In this study, we employ mathematical modeling and monte-carlo sensitivity analysis to explore the working hypothesis that cell-cycle control architectures are HOT networks. If our working hypothesis is true, then fragile cell-cycle mechanisms (reaction steps) should be overrepresented among experimentally observed malfunctions underlying solid and hematological cancers. Moreover, the manipulation of fragile mechanisms in a therapeutic context, which has been suggested by Kitano [29] to be more likely to elicit an efficacious response from a network or system, should also be prevalent in the treatment literature. We test our working hypothesis by computationally screening three overlapping qualitative models of cell-cycle control architectures; we employ monte-carlo sensitivity analysis and k-means clustering to rank-order mechanisms in cell-cycle and then contrast the predicted fragile and robust mechanisms with literature. If cell-cycle control architectures obey the HOT paradigm, then computational identification of fragile mechanisms using protein-protein or protein-DNA network models could be a novel strategy for anti-cancer lead generation or more broadly as a strategy to identify and exploit weakness in arbitrary networks relevant to human health.

## Results

**Figure 1:**
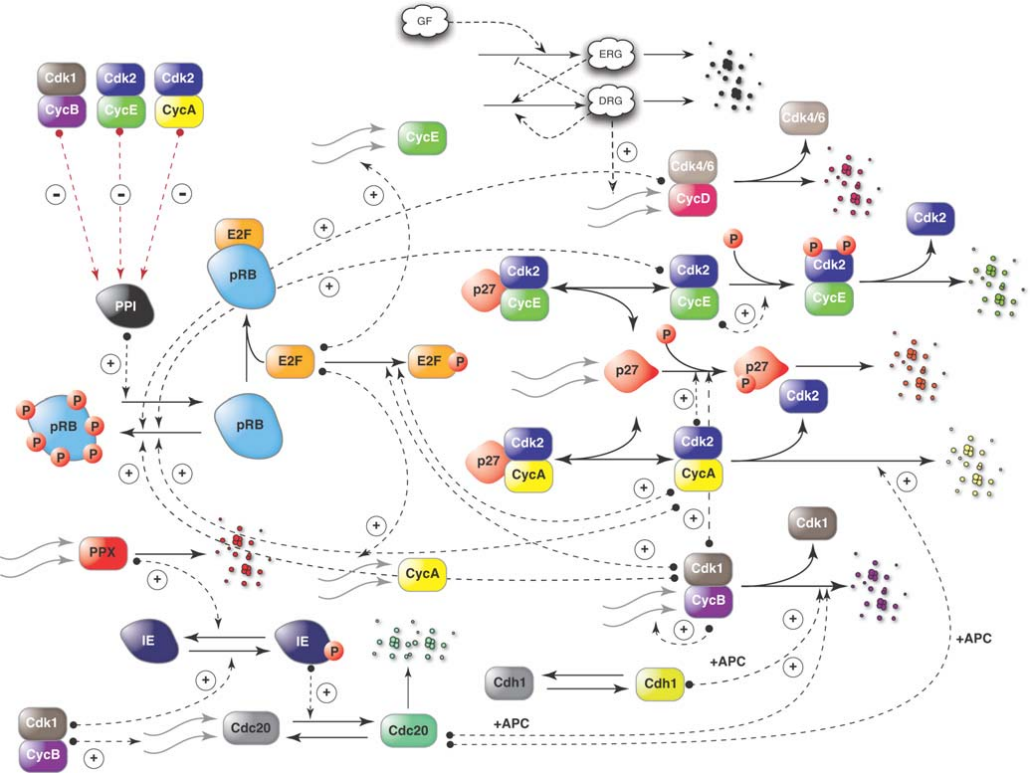
Schematic of the molecular logic of the whole-cycle model of Novak and Tyson [32] used in this study. The Novak and Tyson model, composed of 18 dynamic species, 4 species constraints and 74 parameters, describes both the G1/S and G2/M checkpoints and programmed protein expression and degradation. Nomenclature: Cdk1-Cyclin Dependent Kinase 1, Cdk2 - Cyclin Dependent Kinase 2, Cdk4/6 - Cyclin Dependent Kinase 4 or 6, CycD - Cyclin D, CycB - Cyclin B, CycE - Cyclin E, CycA - Cyclin A, GF - Growth Factor, ERG - Early Response Genes, DRG - Delayed Response Gene, E2F – Transcription Factor E2F, pRB - Retinoblastoma protein, p27 – A Cyclin Dependent Kinase Inhibitor (CKI), also called Kip1, PPI - type1 protein phosphatase, IE - “Intermediary Enzyme”, PPX-A phosphatase inactivating IE , APC - Anaphase Promoting Complex, a family of E3 ligases, Cdh1 - an activator of APC class of ligases, Cdc20 - an activator of APC, Small red circle with P represents a phosphate group, a (+) sign implies positive regulation whereas a (−) sign represents negative regulation.

**Figure 2:**
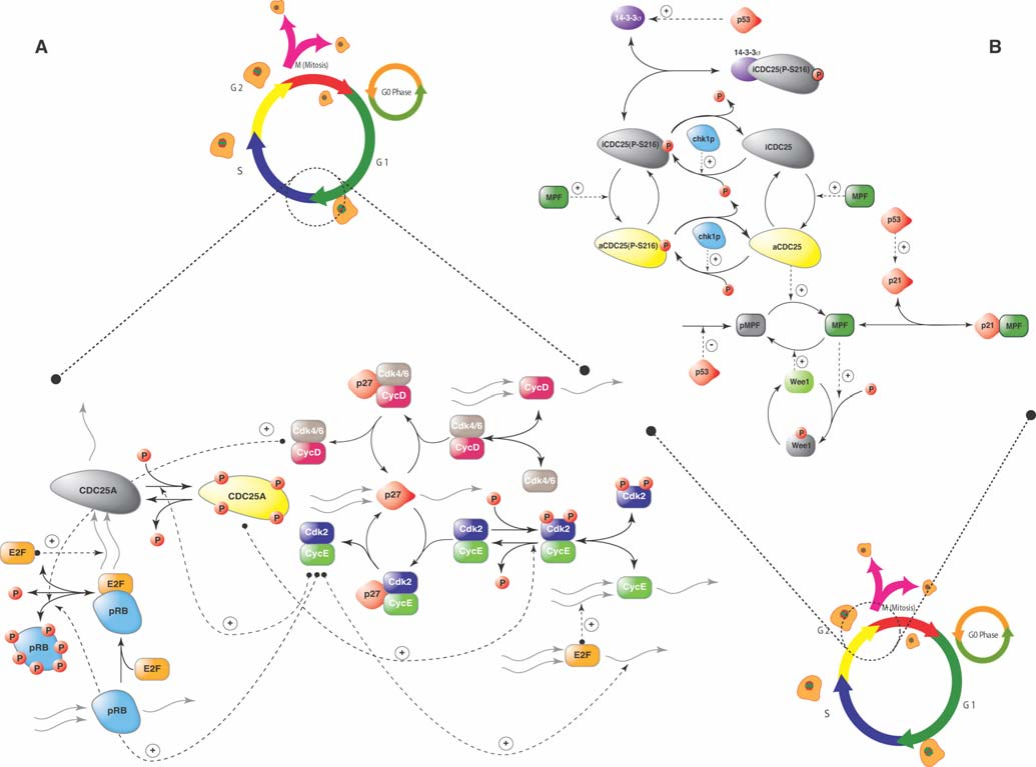
Schematic of the molecular logic of the G1/S (A) and G2/M (B) checkpoint models used in this study. The G1/S model of Qu *et al.*, is composed of 16 dynamic protein balances, 2 species constraints and 44 parameters [31]. TheG2-DNA damage model of Aguda is composed of 15 dynamic protein balances 1constraint and 40 parameters (30). Both the G1/S and G2/M models employ mass action kinetics and the parameters are linear in the mass balances. Nomenclature G1/S: CDC25A - Dual Specificity Phosphatase CDC25A, Cdk2 - Cyclin Dependent Kinase 2, Cdk4/6 - Cyclin Dependent Kinase 4 or 6, CycE - Cyclin E, CycD - Cyclin D, E2F - Transcription Factor E2F, pRB - Retinoblastoma protein, p27 - A Cyclin Dependent Kinase Inhibitor (CKI), also called Kip1. Nomenclature G2/M: pMPF - pre-Maturation Promoting Factor, a complex of CycB (Cyclin B) and Cdk1 (Cyclin Dependent Kinase1) in inactive form, MPF – active form of MPF, aCDC25 - active CDC25 phosphatase, iCDC25 – inactive form of CDC25, aCDC25(P-216) – active CDC25, phosphorylated at Serine 216 residue, iCDC25(P-216) - inactive CDC25, phosphorylated at Serine 216, 14-3-3σ - 14-3-3σ protein. In both the schematics, small red circles with P represent phosphate group, a (+) sign implies positive regulation whereas a (−) sign represents negative regulation.

The whole-cycle model of Novak and Tyson (Fig. 1), the G1-S model of Qu *et al.*, (Fig. 2A) and the G2/M-DNA damage model of Aguda (Fig. 2B) were implemented from literature and screened for fragile mechanisms using monte-carlo sensitivity analysis [30]–[32]. The Novak and Tyson model, which employed a complex description of the G1/S and G2/M checkpoints, programmed protein expression and degradation, was composed of 18 dynamic species, 4 species constraints and 74 parameters. The mass-action G1/S and G2/M-DNA damage models described only the molecular logic in their respective checkpoints; the G1/S model was composed of 16 dynamic protein balances, 2 species constraints and 44 parameters while the G2/M-DNA damage model consisted of 15 dynamic protein balances,1 constraint and 40 parameters. Parameter values for each model were taken from literature. Unreported initial conditions were adjusted so that simulated model trajectories were qualitatively consistent with published values (Supplementary Material Figure S1). The published parameter sets, with fixed initial conditions, were used to generate random parameter sets (N = 500, unless otherwise noted) where each nominal parameter was perturbed by up to ±50%, ±1-order, or ±2-orders of magnitude. Overall State Sensitivity Coefficients (OSSCs) were calculated over the random parameter families for each cell-cycle model using three different numerical algorithms. For each model, the mean OSSC values were ranked-ordered and plotted. The Area Under the Curve (AUC) was used to measure the cumulative sensitivity contribution of each parameter. A cumulative cutoff of 95% of the overall sensitivity was used to establish the list of mechanisms (Supplementary Material Figure S2) which were clustered into three groups (high, medium and low sensitivity) using a k-means algorithm.

Approximately 65% of the G1/S mechanisms (reaction steps) were responsible for 95% of the sensitivity, conversely, the G2-DNA damage network showed a stronger dependence on a few interactions. Of the 44 G1/S reactions steps, 29 were responsible for 95% of the sensitivity (Supplementary Material Figure S2). The distribution of fragility was not specific to any single class of interaction (Table 1). The dephosphorylation of CDC25, the expression of cyclin E, the degradation of the cyclin E-CDK2 complex, and the concentration of the transcription factor E2F were classified as the most fragile reaction steps in the G1/S checkpoint (Table 1, cluster I). A previous model of G1/S by Aguda *et al.*, [33] found that although pRB and cyclin E-CDK2 formed a positive feedback loop, they did not form a sharp robust switch at the restriction point, i.e., the increase in active cyclin E-CDK2 concentration was gradual and sensitive to model parameters. However, addition of CDC25 to the positive cyclin E-CDK2-pRB feedback loop, made the restriction point robust to model parameter variation, thus supporting our findings of the importance of CDC25 interactions. The synthesis, activation and degradation of CKIs, the expression and degradation of CDC25, pRB concentration, the expression of cyclin D and cyclin E-CDK2 mechanisms dominated the second-tier of G1/S fragility (Table 1, cluster II). Tier-three of G1/S fragility involved several cyclin D mechanisms, cyclin E-CDK2 activity and E2F mediated cyclin E expression (Table 1, cluster III). When taken together, the most heavily implicated G1/S protein was cyclin E, with 11 of 29 mechanisms, followed by CKIs with six, CDC25 and cyclin D were each involved in five fragile mechanisms and E2F and pRB were each listed twice. Moreover, 16 of the 29 fragile parameters were functionally associated with cyclin E and cyclin E-CDK2 activity. As expected, the expression and degradation of the G1/S-phase cyclins and their associated CKIs were predicted to be important. However, the expression and degradation of cyclin E and other it's interactions were ranked higher than the corresponding cyclin D mechanisms with the exception of the dissociation of the cyclin E-CDK2-CKI complex. The G2-DNA damage network showed a stronger dependence on a few mechanisms when compared with G1/S; ∼32% or 13 of 40 mechanisms accounted for 95% of the sensitivity (Supplementary Material Figure S2). Consistent with G1/S, no single class of mechanism dominated the fragility list. The most sensitive mechanisms were related to the generation and degradation of the cyclin B-CDK1 complex otherwise known as the Maturation Promoting Factor (MPF) (Table 2). The top five mechanisms were either directly or closely associated with the formation and activity of MPF while mechanisms leading the deactivation of MPF, e.g., the expression, degradation and activity of p21, 14-3-3σ and Wee1 phosphorylation dominated the remaining eight mechanisms (Table 2, cluster III). Activation of inactive MPF complex, whose expression is negatively regulated by p53, was the most sensitive G2 mechanism (Table 2, cluster I), followed by preMPF generation, activation and transport of CDC25 into the nucleus (Table 2, cluster II). The finding that all CDC25 related mechanisms were more fragile than Wee1, is consistent with earlier work by Aguda [34] which showed that even though both Wee1 and CDC25 form a phosphorylation-dephosphorylation (PD) loop with MPF, only CDC25 coupling gave rise to qualitatively different behavior. Interestingly, while the generation of p53 itself was not predicted to be sensitive, interactions involving p53 were prevalent, e.g., the expression of inactive MPF and p21, both of which are regulated by p53, were predicted to be sensitive. Approximately 77% of the Novak and Tyson parameters (57 of 74) were responsible for 95% of the sensitivity (Supplementary Material Figure S2). Both global and local components of the model were predicted to be fragile. The most sensitive global mechanism was the translational efficiency while local mechanisms such as activation of IE (hypothetical protein which activates the E3-ligase CDC20), expression of cyclin B and CDH1 degradation were also predicted to be fragile (Table 3, cluster I). The second-tier mechanisms were associated with deregulation of programmed proteolysis (Table 3, cluster II). Interestingly, while the percentage of mechanisms responsible for 95% of the sensitivity of the Novak and Tyson model was the largest of the three models, several mechanisms in cluster III had small OSSC values, including most of the G1/S checkpoint logic. Thus, sampling the complex Novak and Tyson model produced less information than the mechanistic mass-action based G1/S and G2-DNA damage models.

**Table 1:** Comparison of Overall State Sensitivity Coefficients (OSSC) calculated for the G1/S model of Qu *et al.*, [31].

		OSSC-BDF	OSSC-FD	OSSC-ODE15s
Reaction	Cluster	μ±σ	μ±σ	μ±σ
Dephosphorylation of aCDC25	I	0.6252±0.2980	0.6314±0.2667	0.6942±0.2518
Degradation of aCycE-Cdk2	I	0.5854±0.3452	0.6373±0.3403	0.6756±0.3423
Concentration of E2F	I	0.5710±0.3247	0.6744±0.3062	0.6469±0.2958
Synthesis of CycE	I	0.4583±0.3364	0.5131±0.3476	0.6063±0.3502
Generation of aCKIs	II	0.4513±0.2577	0.5297±0.2540	0.5494±0.2320
Concentration of pRb	II	0.4429±0.2982	0.5224±0.2827	0.5238±0.2725
Phosphorylation of iCDC25	II	0.4442±0.3245	0.4349±0.2905	0.4803±0.2856
Synthesis of iCDC25	II	0.3952±0.1934	0.4535±0.2015	0.4801±0.1690
Synthesis of CycD	II	0.3367±0.2230	0.3984±0.2340	0.4376±0.2411
Formation of iCycE-Cdk2	II	0.3590±0.2275	0.4053±0.2656	0.4271±0.2417
Dephosphorylation of iCKIs	II	0.3841±0.2557	0.4101±0.2428	0.4271±0.2361
Degradation of iCDC25	II	0.3198±0.2129	0.3711±0.2436	0.3789±0.2239
Formation of CycE-Cdk2-CKI	II	0.3410±0.1997	0.3655±0.2106	0.3706±0.1731
Dissociation of CycE-Cdk2 complex	II	0.3023±0.2626	0.3343±0.2946	0.3428±0.3002
Degradation of CycE	II	0.2671±0.2791	0.3163±0.3165	0.3250±0.3262
Phosphorylation of aCKIs	II	0.2909±0.2459	0.2705±0.2017	0.3182±0.2099
Degradation of CKIs	II	0.2678±0.2556	0.2985±0.2803	0.2921±0.2618
Formation of CycD-Cdk4/6	III	0.1987±0.1312	0.2325±0.1410	0.2639±0.1485
Dissociation of CycE-Cdk2-CKI	III	0.2623±0.2512	0.2647±0.2722	0.2585±0.2563
Degradation of CycD	III	0.1867±0.1654	0.2194±0.1786	0.2575±0.1910
iCycE-Cdk2→aCycE-Cdk2	III	0.2096±0.2617	0.2472±0.3047	0.2322±0.2888
Phosphorylation of CDC25 by aCycE-Cdk2	III	0.2057±0.2446	0.2358±0.2828	0.2318±0.2893
Formation of CycD-Cdk4/6-CKI	III	0.1801±0.1130	0.2054±0.1164	0.2268±0.1232
Rate constant for pRb dephosphorylation	III	0.3945±0.3126	0.2016±0.1152	0.2260±0.1164
Degradation of iCKI	III	0.1678±0.1646	0.1644±0.1642	0.2077±0.1815
E2F dependent CycE expression	III	0.2219±0.2849	0.2432±0.3064	0.2064±0.3020
Dissociation of CycD-Cdk4/6-CKI	III	0.1867±0.1654	0.1993±0.1443	0.2046±0.1376
aCycE-Cdk2 regulated pRb phosphorylation	III	0.1551±0.1055	0.1812±0.1122	0.2008±0.1127
Rate constant for CKI phosphorylation	III	0.1638±0.2232	0.1998±0.2602	0.1911±0.2547

Three different numerical methods were used to solve the sensitivity equations; OSSC-BDF: 3rd order fixed step-size backward difference method (implicit); OSSC-FD: forward-finite difference (explicit); and OSSC-ODE15s: 5th order variable step-size backward difference routine (implicit) from the Matlab (The Mathworks, Natick MA) ODE suite. Each member of the nominal parameter set was randomly perturbed by up to ±1-order of magnitude to form a family of random parameter sets (N = 500). OSSC were calculated for every member of the family of random parameter sets. The mean (μ) ±1-standard deviation (σ) are reported.

**Table 2:** Comparison of Overall State Sensitivity Coefficients (OSSC) for the G2-DNA damage model of Aguda [30].

		OSSC-BDF	OSSC-FD	OSSC-ODE15s
Description	Cluster	μ±σ	μ±σ	μ±σ
pMPF→MPF, catalyzed by aCdc25	I	0.8759±0.1475	0.8910±0.1271	0.9924±0.0739
aCdc25→iCdc25	II	0.7676±0.1442	0.7703±0.1181	0.8845±0.0920
Generation of preMPF	II	0.9413±0.1214	0.9720±0.0838	0.8684±0.1130
iCdc25_cyto._→iCdc25_nuc._	II	0.9270±0.1164	0.9417±0.0938	0.8356±0.1014
iCdc25→aCdc25, catalyzed by MPF	II	0.5728±0.2291	0.5010±0.1422	0.2835±0.1517
Generation of p21	III	0.4860±0.1784	0.5031±0.1949	0.2835±0.1517
Degradation of p21	III	0.4833±0.1760	0.4854±0.1838	0.2812±0.1481
p21+MPF→p21−MPF	III	0.3382±0.1406	0.3413±0.1504	0.2017±0.1248
p21−MPF→p21+MPF	III	0.3352±0.1373	0.3254±0.1438	0.1979±0.1172
Generation of 14-3-3σ protein	III	0.3434±0.1250	0.3802±0.1459	0.1913±0.1060
Degradation of 14-3-3σ protein	III	0.3421±0.1247	0.3625±0.1390	0.1909±0.1059
Wee1→Wee1P, catalyzed by MPF	III	0.3214±0.1338	0.3274±0.1489	0.1739±0.0878
Wee1P→Wee1	III	0.3078±0.1306	0.2993±0.1381	0.1666±0.0855

Three different numerical methods were used to solve the sensitivity equations; OSSC-BDF: 3rd order fixed step-size backward difference method (implicit); OSSC-FD: forward-finite difference (explicit); and OSSC-ODE15s: 5th order variable step-size backward difference routine (implicit) from the Matlab (The Mathworks, Natick MA) ODE suite. Each member of the nominal parameter set was randomly perturbed by up to ±1-order of magnitude to form a family of random parameter sets (N = 500). OSSC were calculated for every member of the family of random parameter sets. The mean (μ) ±1-standard deviation (σ) are reported.

**Table 3:** Comparison of Overall State Sensitivity Coefficients (OSSC) for the whole-cycle model of Novak and Tyson [32].

		OSSC-BDF	OSSC-FD	OSSC-ODE15s
Description	Cluster	μ±σ	μ±σ	μ±σ
Translational efficiency (▭)	I	0.7904±0.3264	0.8647±0.2372	0.6657±0.3816
Activation of ‘IE’ (k_31_)	I	0.6026±0.3071	0.6026±0.3071	0.5361±0.3843
Generation of CycB (k_1_)	I	0.5650±0.3015	0.4993±0.2299	0.5002±0.3471
Cdh1 degradation (k_4_)	I	0.4043±0.2443	0.3805±0.2361	0.4997±0.3765
Degradation of ‘IEP’ (k_32_)	II	0.4958±0.2863	0.3576±0.2219	0.4759±0.3417
Generation of Cdh1 (k′_3_)	II	0.4434±0.2863	0.3576±0.2219	0.4759±0.3417
CycA mediated degradation of Cdh1 (γ_A_)	II	0.4434±0.2934	0.3021±0.1895	0.4482±0.3853
Degradation of ‘PPX’(k_34_)	II	0.2266±0.1621	0.2788±0.1995	0.2835±0.2604
Generation of dephosphatase PPX (k_33_)	III	0.2224±0.1652	0.2152±0.1616	0.2572±0.2240
Activation of Cdc20 (k_13_)	III	0.2441±0.3096	0.2557±0.2616	0.2202±0.2782
CycE dependent CycE∶Kip1 dissociation (k_8_)	III	0.0463±0.0798	0.0041±0.0058	0.1989±0.2545
CycE∶Kip1 dissociation giving Kip1 (k′_8_)	III	0.0463±0.0798	0.0105±0.0635	0.1988±0.2545
CycE dependent Kip1 accumulation (ψ_E_)	III	0.0438±0.0744	0.0078±0.0371	0.1861±0.2386
Cdh1 dependent degradation of Cyc B (k′_2_)	III	0.1143±0.1189	0.0865±0.1405	0.1759±0.1690
Generation of Cyc B (k″_1)_	III	0.1486±0.0760	0.1333±0.0672	0.1751±0.1649
Degradation of Cdc20 (k_14_)	III	0.2402±0.2678	0.1898±0.2238	0.1692±0.2057
Total E2F (E2F_T_)	III	0.1460±0.1282	0.4249±0.2385	0.1524±0.1424
Degradation of DRGs (k_18_)	III	0.0463±0.1020	0.0003±0.0006	0.1461±0.1720
Expression of CycA, catalyzed by aE2F (k_29_)	III	0.1697±0.1366	0.2639±0.1444	0.1334±0.1525
aE2F (k_7_) mediate CycE expression	III	0.0367±0.0625	0.0035±0.0038	0.1325±0.1489
Formation of ‘GM’ (k_27_)	III	0.0911±0.1014	0.1276±0.0863	0.1307±0.1474
Degradation of Cdc20 (J_4_)	III	0.0649±0.0743	0.0743±0.1348	0.1281±0.1331
CycB dependent degradation of Cdh1 (γ_B_)	III	0.0902±0.1296	0.0478±0.0563	0.1281±0.1331
Synthesis of p27^Kip1^ (k_5_)	III	0.0272±0.0447	0.0032±0.0041	0.1274±0.1232
Synthesis of DRG products (k_17_)	III	0.0442±0.0992	0.0003±0.0005	0.1205±0.1754
Maximum specific growth rate (μ)	III	0.1231±0.1431	0.1724±0.1096	0.1176±0.1490
CycE dependent decrease in Kip1 (k_6_)	III	0.0264±0.0432	0.0030±0.0038	0.1161±0.1161
Decrease in E2F (k_23_)	III	0.0713±0.0734	0.2138±0.2094	0.1130±0.1080
Degradation of Cdc20 (k_12_)	III	0.0686±0.0969	0.0308±0.0419	0.1091±0.1041
Degradation of free E2F (aE2F (k_22_))	III	0.0683±0.0969	0.0308±0.0419	0.1091±0.1041
Total PP1_T_ (PP1_T_)	III	0.0220±0.0394	0.0001±0.0002	0.1011±0.1034
Synthesis of CycB (J_1_)	III	0.0577±0.0356	0.0594±0.0378	0.0999±0.1341
Degradation of ‘GM’ (k_28_)	III	0.0841±0.0957	0.1025±0.0688	0.0961±0.1092
CycE/A activation of PP1 (K_21_)	III	0.0206±0.0370	0.0001±0.0002	0.0945±0.0972
Cdh1 dependent CycB degradation (k_2_)	III	0.0706±0.0676	0.0547±0.0447	0.0911±0.1137
CycD dependent E2F∶Rb dissociation (k_20_)	III	0.0224±0.0402	0.0022±0.0208	0.0878±0.0941
CycE dependent activation of PP1 (φ_E_)	III	0.0183±0.0324	0.0011±0.0104	0.0865±0.0911
Degradation of ‘IEP’ (J_32_)	III	0.0467±0.0551	0.0313±0.0428	0.0853±0.0774
Degradation of CycD and CycD∶Kip1 (k_10_)	III	0.0174±0.0307	0.0002±0.0004	0.0852±0.0889
GF dependent synthesis of CycD (k_9_)	III	0.0171±0.0304	0.0012±0.0104	0.0805±0.0874
Degradation of ERG (k_16_)	III	0.0129±0.0227	7.194×10^−7^±1.728×10^−6^	0.0802±0.0912
Total pRb concentration (Rb_T_)	III	0.0179±0.0321	0.0068±0.0530	0.0780±0.0817
PP1 dependent pRb activation (k_19_)	III	0.0179±0.0321	0.0001±0.0002	0.0772±0.0807
CycB dependent Cdc20 formation (k_11_)	III	0.0138±0.0230	0.0023±0.0171	0.0770±0.0826
Formation of Cdh1 (J_3_)	III	0.0153±0.0224	0.0118±0.0136	0.0710±0.1542
Formation of CycE-Cdk2-Kip1 (k_25_)	III	0.0142±0.0246	0.0004±0.0006	0.0695±0.0687
Cdc20 dependent CycB degradation (k″_2_)	III	0.0697±0.1035	0.0489±0.0944	0.0685±0.1210
Formation of ERGs (k_15_)	III	0.0124±0.0222	8.593×10^−7^±2.233×10^−6^	0.0662±0.0759
DRG dependent formation of ERG(J_15_)	III	0.0208±0.0399	4.998×10^−7^±8.465×10^−7^	0.0649±0.0997
CycB dissociation of CKI complex(η_B_)	III	0.0170±0.0328	0.0003±0.0004	0.0598±0.0787
CycD-Cdk4/6-Kip1 association(k_24_)	III	0.0110±0.0199	0.0002±0.0004	0.0469±0.0563
CycE dissociation of CKI complex(η_E_)	III	0.0099±0.0134	0.0021±0.0022	0.0461±0.0510
Cdh20 depdendent Cdh1 formation (k_3_)	III	0.0732±0.1007	0.0607±0.1165	0.0439±0.0851
CycE dependent pRb phosphorylation (λ_E_)	III	0.0098±0.0173	7.812×10^−5^±1.044×10^−4^	0.0413±0.0467
Cyclin dependent pRb phosphorylation (k_26_)	III	0.0112±0.0199	0.0001±0.0002	0.0383±0.0470
CycB dependent pRb phosphorylation (λ_B_)	III	0.0123±0.0238	5.447×10^−5^±1.567×10^−4^	0.0333±0.0515
Cdc20 dependent CycA degradation (k_30_)	III	0.0182±0.0289	0.0138±0.0184	0.0329±0.0458

Three different numerical methods were used to solve the sensitivity equations; OSSC-BDF: 3rd order fixed step-size backward difference method (implicit); OSSC-FD: forward-finite difference (explicit); and OSSC-ODE15s: 5th order variable step-size backward difference routine (implicit) from the Matlab (The Mathworks, Natick MA) ODE suite. Each member of the nominal parameter set was randomly perturbed by up to ±1-order of magnitude to form a family of random parameter sets (N = 150). OSSC were calculated for every member of the family of random parameter sets. The mean (μ) ±1-standard deviation (σ) are reported.

**Figure 3:**
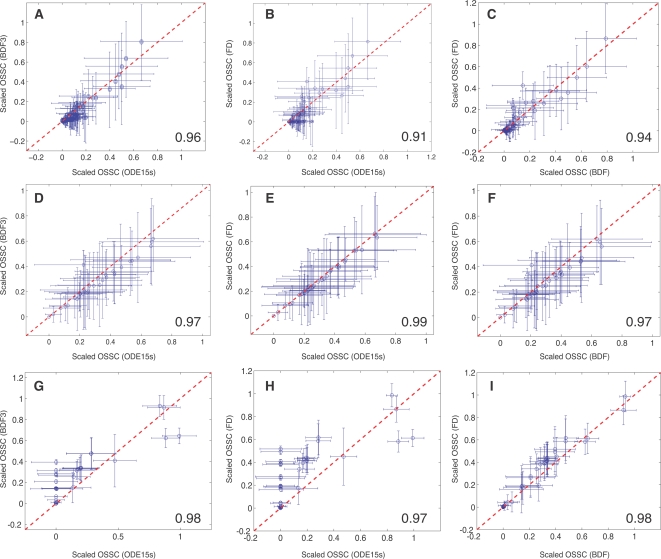
Sensitivity analysis results as a function of model and numerical method. Scaled Overall State Sensitivity Coefficients (OSSC) were calculated for each cell-cycle model over a family of random parameters sets (N = 500 unless otherwise noted) generated by randomly perturbing the published set by ±1-order of magnitude. Three different numerical methods were used to solve the sensitivity equations to control for numerical artifacts. A–C: Sensitivity results for the Novak and Tyson model [32]. D–F: Sensitivity results for the G1/S checkpoint model of Qu *et al.*, [31]. G–I: Sensitivity results for the G2/M-DNA damage model of Aguda [30]. The different numerical techniques used to solve the sensitivity equations yield qualitatively similar results as quantified by the Spearman rank correlation between any two methods (lower right-hand corner of each plot).

**Figure 4:**
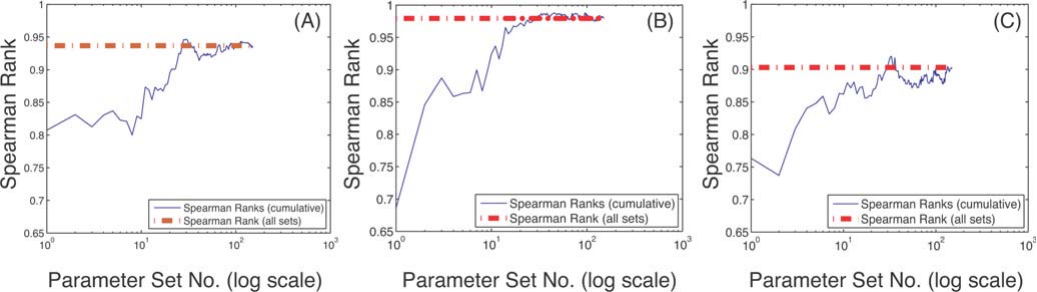
Effect of the parameter perturbation size on conclusions drawn from sensitivity analysis of the G1/S model. A family of random parameter sets was constructed (N = 150) from the nominal set, where each parameter was perturbed by upto ±50%, ±1-order or ±2-orders of magnitude. The ODE15s routine of Matlab (The Mathworks, Natick MA) was used to solve the sensitivity equations. A: Cumulative Spearman ranks between parameters sets with ±50% change and ±1order change. B: Cumulative Spearman ranks between parameters sets with ±1-and ±2-orders of magnitude change. C: Cumulative Spearman ranks between parameters sets with ±50%-and ±2-orders of magnitude change.

**Figure 5:**
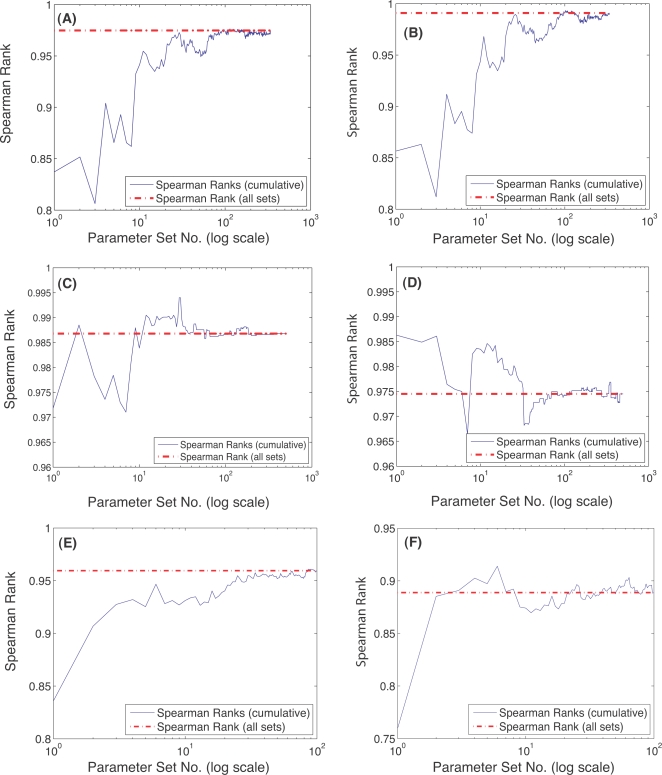
Spearman rank correlation as a function of the number of random parameter sets sampled. The red-dashed line in all cases denotes the cumulative Spearman Rank obtained by sampling all parameter sets for any two methods. A–B: Cumulative Spearman rank versus the number of parameter sets sampled for the G1-S model using the BDF3 and ODE15s methods (A) and Finite Difference (FD) and ODE15s methods (B), respectively. C–D: Cumulative Spearman rank versus the number of parameter sets sampled for the G2-M model using the BDF3 and ODE15s methods (C) and Finite Difference (FD) and ODE15s methods (D), respectively. E–F: Cumulative Spearman rank versus the number of parameter sets sampled for the whole-cycle model using the BDF3 and ODE15s methods (E) and Finite Difference (FD) and ODE15s methods (F), respectively. In all models and numerical methods, the cumulative Spearman rank converges to population value, however, the rate of convergence, i.e., the number of random sets required to be sampled, is different for each model and method.

The qualitative conclusions drawn from sampling the cell-cycle models were robust to the choice of solution method and the size of the parameter perturbation but sensitive to the number of parameter sets sampled. Three different numerical techniques were used to solve the sensitivity equations to control for possible numerical artifacts. The ODE15s routine of Matlab (The Mathworks, Natick MA), a third-order backward-difference implicit method (BDF3; see Supplementary Material S1) and forward finite difference (FD), generated qualitatively similar sensitivity results (Fig. 3). The lowest Spearman rank between any two methods (ODE15s versus FD for the G1/S model) was 0.91 indicating a worse case correlation of approximately 91%. Interestingly, while the Spearman rank indicated good agreement between the solution methods, there were statistically significant shifts in OSSC values indicating the solution methods systematically shifted mechanisms, i.e., different OSSC values were calculated but the order or ranking of mechanisms was maintained (see Supplemental Material Table S1). Two additional sampling controls were conducted to verify the robustness of the qualitative conclusions drawn from our analysis. First, the perturbation size used to generate the random parameter families was varied to test if different conclusions would have been drawn with different perturbation sizes; OSSC values computed over random parameter families generated using ±50%, ±1-order and ±2-orders of magnitude showed no qualitative difference as quantified by the Spearman rank correlation for the G1/S model (Fig. 4). The worst case correlation of 0.90 was observed between the ±50% and ±2-orders of magnitude cases indicating on average 90% of the conclusions drawn between the two cases were consistent (Fig. 4C). Such a strong correlation in Spearman ranks across 2-orders of magnitude in the parameter values might suggest that network structure (connectivity) is more important than parameter values. Comparison of exactly similar mechanisms across the three models supported the hypothesis of connectivity dominance where mechanisms classified as either fragile or robust in the G1/S and G2-DNA damage models were also predicted to be important in the Novak and Tyson model, albeit with different ranks (Table 4). There were 11 mechanisms which appeared exactly in each model, 10 mechanisms were classified similarly while one was ranked inconsistently. Second, the cumulative Spearman rank correlation between sensitivity results generated using the ODE15s, BDF3 and FD methods for each model was calculated as a function of the number of parameter sets sampled. While the cumulative Spearman rank converged to the population mean as the number of parameter sets increased, a population size dependence was observed (Fig. 5). For each model, the results reported were obtained in the region of convergence; hence, no new information would have been gained if additional random parameter sets were sampled.

**Table 4:** Comparison of OSSC ranks for common mechanisms in the G1/S, G2-DNA damage and Novak and Tyson models.

Mechanism	G1/S (%)	G2/M (%)	Whole-cell model (%)
Generation of preMPF	-	93±2	80±18
**Total concentrations**			
Total E2F concentration	93±15	-	77±10
Total pRb concentration	86±15	-	43±16
**Reactions of CKIs**			
Generation of CKIs	86±10	85±2	68±12
CycE-Cdk2 associating with CKI	70±9	-	38±15
Dissociation of CycE-Cdk2-CKI	57±19	-	(*8±23*, 5±19)
CycD-Cdk4/6 associating with CKI	48±8	-	31±19
Dissociation of CycD-Cdk4/6-CKI	39±11	-	(47±14, *8±23*, 4±19)
**Generation and Degradation**			
Degradation of CycE	66±24	-	38±15
Degradation of CycD	55±15	-	47±14
CycE generation catalyzed by E2F	41±26	-	73±16

The mean percentage ranking, defined as the fractional distance from the lowest ranked mechanism, ±1-standard deviation is reported. The 95% cutoff for mechanisms to be included in the fragile set was 34%, 68% and 23% for the G1/S, G2-DNA damage and Novak and Tyson models, respectively.

## Discussion

Literature evidence supports the hypothesis that computationally identified fragile cell-cycle interactions represent efficacious targets. Consider the fragility of CDC25 mechanisms. Boutros *et al.*, recently reviewed the role of CDC25 phosphatases and CDC25 inhibitors in human cancer progression and treatment [35]. While the inhibition of CDC25 as a cancer treatment strategy is still in the laboratory stage, several CDC25 inhibitors in development have shown promising results. The CDC25 inhibitor PM20 inhibited growth in human hepatoma-derived Hep3B cell-lines at a inhibitory concentration (IC) >700 nM, PM-20 also inhibited the growth of several other cell-lines, albeit at higher ICs [36]. BN82685, which inhibited CDC 25A, B and C *in-vitro* and *in-vivo* and repressed the growth of HeLa and human pancreatic tumor Mia PaCa-2 xenografts in athymic nude mice, also inhibited the growth of human cell lines resistant to cytotoxic drugs e.g., the human myeloblastic leukemia cell-line HL-60 [37]. The CDC25 antagonist, CPD-5, inhibited the growth of the rat hepatoma cell-line JM-1 *in-vitro* and the mouse cancer cell-line tsFT210 through selective inhibition of CDC25 [38]. Thus, inhibition of CDC25 represents a viable treatment option which could be pursued further in the clinic. Inhibition and degradation of the active cyclin E-CDK2 complex, the second ranked mechanism in the G1/S network, has also been exploited as a treatment strategy. Bristol-Myers Squibb (BMS) developed BMS-387032, a cyclin E-CDK2 inhibitor, with an IC50 of 95 nM [39]. Preclinical and phase I ovarian cancer studies demonstrated that BMS-387032 possessed better efficacy than Flavopiridol, a promiscuous CDK inhibitor [40]. Flavopiridol, the first cyclin dependent kinase inhibitor in clinical trials, alone or in combination with other drugs is currently being investigated in 52 active phase I or II trials [41]. Flavopiridol has been proposed for the treatment of recurrent, locally advanced, or metastatic soft tissue sarcoma [42], lymphoma and multiple myeloma [43], metastatic breast cancer (with Trastumuzumab) [44] or in combination with other drugs (Cisplatin and Carboplatin) for the treatment of advanced solid tumors [45]. Cyclin E expression, the fourth ranked mechanism in the G1/S model, has also been explored therapeutically for the treatment of pancreatic and lung cancers [46], [47]. The correlation between fragility and treatment strategy was also found to hold for the G2/M-DNA damage network. The activation of preMPF (cyclin B–CDK1 complex), catalyzed by CDC25, was predicted to be the most sensitive mechanism in the G2/M-DNA damage model while three of the four tier-two G2/M-DNA mechanisms were associated with CDC25 activity. Bryostatin-1, a protein kinase C (PKC) inhibitor and antagonist of the cyclin B-CDK1 complex, has been explored in the clinic for the treatment of multiple myeloma [48], relapsed non-Hodgkin's lymphoma and chronic lymphocytic leukemia [49]. In preclinical models, Bryostatin-1 has demonstrated single-agent activity against B16 melanoma, M5076 reticulum sarcoma and L10A B-cell lymphoma [50] and has been shown to disrupt cyclin B-CDK1 complex formation and activity by several different mechanisms [51], [52]. When taken together, the top fragile mechanisms for both the G1/S and G2/M phases of the cell-cycle, estimated by monte-carlo sensitivity analysis, were found to be consistent with on-going preclinical and clinical trials for the treatment of a broad spectrum of human cancers.

Modulation of translational efficiency and the manipulation of programmed proteolysis, prominently featured among the group of fragile mechanisms across all the models, are also active areas of therapeutic development. Initiation of translation in eukaryotes is thought to be rate limiting [53] and overexpression of initiation components, for example the initiation factor elF4E, occurs frequently in human cancers [54]. Arnqvist and coworkers explored translation inhibition in MCF-7 breast cancer cells following cycloheximide, puromycin or emetine exposure in the presence and absence of Insulin-like Growth Factor1 (IGF-1) [55]. Addition of puromycin, cycloheximide and emetine in the absence of IGF-1 resulted in increased apoptosis at 48 hr relative to the control, however, when IGF-1 was present, a concentration dependent reduction in apoptosis was observed. Bjornsti and Houghton recently reviewed another small molecule translation inhibitor, Ramapycin [56], which inhibits the Target of Ramapycin (TOR) protein, a serine/threonine kinase involved in translation and other functions. While Ramapycin has FDA approval as an immunosuppressant, development of anticancer therapies has been slow despite anti-tumor activity against established solid-tumor models [57], [58]. Ramapycin analogs have been evaluated in clinical trials for the treatment of different indications including pediatric patients with relapsed or refractory acute leukemia and renal-cell carcinoma [56], [59]. Peptide inhibitors have also been used to downregulate translation e.g., BL22, an immunotoxin developed for the treatment of Chronic Lymphocytic Leukemia (CLL) [60], consists of the variable FV fragment of the RFB4 antibody conjugated to the anti-translation peptide PE38. The second group of fragile mechanisms predicted in Novak and Tyson and more generally across the G1/S and G2/M-DNA damage networks involved deregulation of programmed protein degradation. Programmed proteolysis via the Ubiquitin Proteasome System (UPS), a critical component driving cell-cycle progression [61], has been the target of several different therapeutic developments [62]. The ubiquination of target proteins involves the coordinated activity of the ubiquitin activating enzyme family (E1), the ubiquitin-conjugating enzyme family (E2) and the ubiquitin ligase family (E3) [63]. While E1 malfunctions have not been observed in cancer, deregulation of E3 and to a lesser extent E2 activity has been directly linked to cancer progression [63]. The Novak and Tyson model has only a skeleton representation of UPS, however, it does explicitly represent Cell Division Cycle protein 20 (CDC20), CDH1 and Anaphase Promoting Complex/Cyclosome (APC/C), all of which are E3 components. APC/C is the core subunit to which the adapter proteins CDC20 and CDH1 bind [64]–[66]. Inhibition of specific E3 ligases remains a technical challenge [67], however, cis-imidazoline analogs called Nutlins have been developed which inhibit MDM2, an E3-ligase responsible for the recognition of p53. Activity of Nutlins-3 against a human osteosarcoma xenograft model in nude mice showed 90% inhibition of tumor growth relative to control [68].

While multiple lines of experimental evidence support the assertion that malfunctions in fragile mechanisms are implicated in solid and hematological cancers, some evidence is contradictory. CDC25 activity, cyclin E expression and activity of cyclin E-CDK2 were the largest group of fragile G1/S mechanisms. Traditionally, cyclin E expression and cyclin E-CDK2 activity were thought to be critical for cell-cycle progression [69]. Ohtsubo *et al.*, have shown that cyclin E-CDK2 activity was maximum during the G1/S phase and overexpression of cyclin E accelerated cell-cycle progression [70]. Lucas *et al.*, showed that abnormal cyclin E but not Cyclin D1 expression was able to override G1 arrest by the INK4a family of CKIs [71]. Keyomarsi *et al.*, found that cyclin E expression plays a strong role in human breast cancer tumors and the cyclin E-CDK2 complex remains active throughout the cell-cycle suggesting the now established hypothesis that truncated (deregulated) cyclin E variants were responsible for the constitutive function of cyclin E-CDK2 in breast cancer tumors [72], [73]. Recent studies, however, have challenged the traditional role of cyclin E. Deletion of both cyclin E genes was lethal in-utero but deletion of cyclin E1 or cyclin E2 was tolerated with no obvious abnormalities [74]. Interestingly, double cyclin E knockout mice were born alive if cyclin E was restored in the embryonic component of the placenta [74] and CDK2 null mice were born viable and healthy [75]. Thus, while the cyclin E and CDK2 knockout studies seem to contradict the essential role of cyclin E, clinical evidence suggests further studies are required. Evidence supporting the involvement of other fragile components, such as the concentration of E2F and pRB (constraints in the G1/S and Novak and Tyson models), is also prevalent in the literature [76], [77]. However, contradictory evidence suggests that the role of cyclin D mechanisms maybe complex. Sensitivity analysis suggested that cyclin D-CDK4/6 and cyclin D-CDK4/6-CKIs trimer mechanisms were robust or only moderately sensitive while cyclin D expression was fragile in the G1/S checkpoint. While Keenan *et al.*, demonstrated in IIC9 Chinese hamster embryonic fibroblasts that cyclin E expression renders cyclin D-CDK4 dispensable [78], overexpression of cyclin D variants, particularly cyclin D1, has been observed in several human cancers [79], [80]. Moreover, cyclin D1, D2 or D3^−/−^ mice displayed tissue specific phenotypes including defective proliferation [81]–[83]. However, while mice lacking all the cyclin D genes died by day E17.5 of gestation, most tissue and organs were formed by day E13.5 indicating that cyclin D was not required for embryogenies [84]. When taken together, the retrospective cyclin E studies in breast cancer patients and the CDC25 studies support the hypothesis that malfunctions in fragile mechanisms are strongly implicated in cancer progression. However, the cyclin E and CDK2 knockout studies as well the confusing role of cyclin D suggests a more nuanced perspective in which redundant proteins or subsystems might be able to compensate for malfunctions in fragile mechanisms.

Consistent with the conjecture of Kitano, the anecdotal comparison between predicted fragile mechanisms and literature suggested that cell-cycle control architectures are HOT networks [29]. However, while different controls were conducted to ensure the fidelity of the monte-carlo sampling protocol, the mathematical models being explored were coarse-grained and not structurally complete. While quantifying the impact of structural uncertainty remains a critical challenge, the general correlation between efficacy and fragility appears to be model independent as other studies have yielded similar results [19]. Moreover, initial results presented here suggest that while the quantitative values of sensitivity coefficients calculated using different models with overlapping biology will change between models, the qualitative conclusions drawn may be invariant. However, this conclusion is likely true only for a subset of mechanisms. One possible strategy to explore structural uncertainty would be to construct detailed subsystem models of the coarse-grained components which were determined by our analysis to be fragile, e.g., translation or UPS. While this top-down strategy does not specifically address structural uncertainty, it does allow us to determine the molecular interactions which are perhaps mediating fragility in the coarse-grained model. A second critical issue is the choice of sensitivity metric. OSSCs quantify the overall impact that a parameter has; however, other measures of sensitivity might be better suited for analysis of the cell-cycle. Doyle and colleagues have established tools for the analysis of mammalian circadian rhythm that could prove useful in understanding how fragility influences phenotypic properties such as division frequency [17], [85], [86]. A third critical issue not addressed in this study was safety. Highly efficacious strategies have resulted in unwanted and possible harmful side effects, e.g., the association of rofecoxib with adverse cardiovascular events [87]. While there may not be an obvious linkage between fragility and safety for single agents, initial retrospective studies by Luan *et al.*, using combinations of coagulation inhibitors, have suggested that shifts in mechanism rank could be used to understand molecular drug-drug synergies [19].

## Materials and Methods

### Model formulation and sensitivity analysis

The cell-cycle models used in this study [30]–[32] were represented as systems of Differential Algebraic Equations (DAEs):
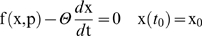
(1)where x∈*R^m^* denotes the concentration vector, f(x, p)∈*R^m^* denotes the mass balance equation vector describing the kinetics and connectivity of the cell cycle network and p∈*R^p^* denotes the parameter vector. The diagonal *m*×*m* matrix Θ contains 1's for dynamic elements of the concentration vector, 0 otherwise (constraints).

The fragile elements of the cell-cycle networks were determined by computing Overall State Sensitivity Coefficients (OSSC) [17]. OSSC values were calculated by first calculating the first-order sensitivity coefficients (at time t_k_):
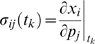
(2)which are solutions of the equation:
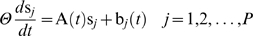
(3)subject to the initial condition s*_j_*(*t*_0_) = 0. The quantity *j* denotes the parameter index, *P* denotes the number of parameters and s*_j_* denotes the *m*×1 vector of first-order sensitivity coefficients with respect to parameter *j*. The Jacobian matrix (***A***) and the matrix of first derivatives of the mass balances w.r.t the parameter values (***B***) (whose columns are denoted by b*_j_*) are given by:
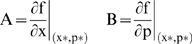
(4)where ***x*** denotes a point along the *nominal* or *unperturbed* system solution. We solved the sensitivity equations for each parameter using three different numerical methods to control for possible artifacts; a 3-order Backward Difference (BDF3) method was compared with forward Finite Difference (FD), and the fifth-order variable step-size ODE15s routine of Matlab (The Mathworks, Natick MA). The matrices ***A*** and ***B*** were estimated numerically at each time step using a generalized gradient algorithm [88]. Overall State Sensitivity Coefficients (OSSC), first used by Stelling *et al.*, to characterize mechanisms in circadian rhythm as fragile or robust [18], were calculated for each parameter *j*:
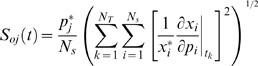
(5)The quantity *N_T_* denotes the number of time points used in the simulation while *N_s_* denotes the number of proteins/protein complexes in the model. To account for parametric uncertainty, the OSSC values (*S_oj_*) were calculated over a family of random parameter sets; we randomly perturbed each nominal parameter by up to ±1-order of magnitude then solved the sensitivity balances for each family member. To control for perturbation effects, two other random parameter families were also tested (±50% and ±2-orders of magnitude, N = 500).

### Statistical and clustering analysis of OSSC values

Three different tests were performed to identify large statistically significant shifts in the OSSC values. The OSSC values calculated over the family of parameter sets were assumed to be normally distributed. The statistical significance of shifts in OSSC values for each algorithm relative to ODE15s (control) were determined by performing a Welch t-test with the null hypothesis that the means of the OSSC values were equal at a 1% significance level [89]. The list of significant OSSC values was further restricted to only those shifts with a magnitude larger than a specified z-score (1.0) away from the squared mean displacement over the significant OSSC values. We defined the displacement of an OSSC value relative to the control as:
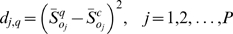
(6)where 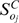 denotes the mean OSSC value over the family of parameter sets for parameter *j* in the control while 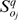 denotes the same quantity for algorithm *q*. A significant shift in OSSC value was accepted if:
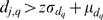
(7)where ***z*** denotes a desired z-score, 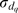 denotes the standard deviation of the total displacement over all significant OSSC values for the *q^th^* numerical algorithm and 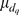 denotes the mean of the significant displacements for algorithm *q*. Large statistically significant shifts in OSSC values, while perhaps indicative of the shifting importance of mechanisms, do not guarantee that mechanisms are qualitatively different between the algorithms considered (see Supplementary Material Table S1). The Spearman rank correlation denoted by ρ and defined as:
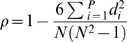
(8)was used to measure the difference in qualitative ranking of mechanisms between algorithms considered. The quantity *d_i_* denotes the difference in the ordinal rank of mechanisms between algorithms or perturbation size, *N* denotes the number of pairs of values and *P* denotes the number of parameters considered. The Spearman rank is bounded by −1≥ρ≥1; a Spearman rank of one indicates that two ranked lists are identical, a Spearman rank of negative one indicates a perfect negative correlation, while a Spearman rank of zero indicates that two ranked lists are uncorrelated.

The distributions of OSSC values obtained from monte-carlo sampling were clustered using a k-means algorithm [90]. The mean and standard deviation obtained from the monte-carlo sensitivity analysis was used to estimate the underlying OSSC distribution (N = 500 points) where the OSSC values were assumed to be normally distributed. One-hundred different clustering attempts were run for each model to control for clustering artifacts. The most probable configuration was reported.

## Supporting Information

Material S1 (1.07 MB DOC)

Figure S1 Qualitative comparison of simulations results of the model implementations used in this study. A–B: Free and bound Cyclin E versus time for the reimplementation (A) and published (B) the G1/S model of Qu et al., [31]. C–D: Concentration profiles of the Wee1, MPF and active CDC25 proteins versus time for the reimplementation (C) and published (D) G2/M DNA damage model of Aguda [30]. E–F: Concentration profiles for the Cdh1 protein and the Cdk1:CycB complex versus time for the reimplementation (E) and published whole-cycle model of Novak and Tyson [32]. In all cases the reimplemented models were qualitatively consistent with published results.
(1.95 MB EPS)

Figure S2 Cumulative Sensitivity as a function of parameter rank. The cumulative sensitivity contribution of each parameter was calculated by calculating the Area Under the Curve (AUC) using the trapazoid rule. Mechanisms responsible for 95% of the total sensitivity in each model were collected, clustered and analyzed. Panel A shows the result for G1/S model, Panel B - G2/DNA damage model and Panel C shows the plot for the whole cell model.
(0.33 MB EPS)

Table S1 Statistically significant shifts of Overall State Sensitivity Coefficients (OSSCs) between solution methods computed using the Welch t-test. The mean and one standard deviation of the OSSC score computed over the family of random parameter sets is reported. Only shifts recorded with a p-value of 0.01 and z-score of 1 are shown.
(0.04 MB DOC)
