# Supplementary material for: A Test of Highly Optimized Tolerance Reveals Fragile Cell-Cycle Mechanisms Are Molecular Targets in Clinical Cancer Trials
Source: PLoS One. 2008 Apr 23;3(4):e2016. doi: 10.1371/journal.pone.0002016 (PMC2291571; doi:10.1371/journal.pone.0002016)
Supplement: Material S1 — (1.07 MB DOC) [file pone.0002016.s001.doc]

Supplemental Material

**Calculation of OSSCs using BDF-3**. The sensitive or fragile elements of the cell cycle architecture were determined by computing Overall State Sensitivity Coefficients (OSSC) using three different numerical methods, a forward Finite Difference (FD), the ODE15s routine of Matlab (The Mathworks, Natick MA) and a third-order Backwards Difference Formula (BDF3). BDF3 is an implicit, fixed step size method for solving ODEs numerically. OSSC values were calculated by first calculating the first-order sensitivity coefficients.

(S1)

which are the solutions of the matrix differential equation

(S2)

subject to the initial condition where denotes the number of parameters in the model. Here is a column of the matrix. The Jacobian matrix () and the matrix of first derivatives of the mass balances w.r.t the parameter values () were calculated as:

(S3)

Where denotes a point along the nominal or unperturbed system solution and denotes the mass balances equations. In general, when the – order BDF formula is substituted in the Eqn (S2), we get:

(S4)

where

(S5)

S-1

(S6)

The quantities and are constants specific to a particular BDF method, *k* represents a time-point and is the time-step. The calculation of **A** and **B** at the time point, i.e., and was made efficient by reformulating the estimation of the Jacobian and **B** matrix as a least squares problem, i.e., elements of were computed using

(S7)

and the elements of B k+1 by

(S8)

where and represent the vectors having values slightly perturbed from the nominal set and represent the rows of and respectively.

**Welch t-test results and shifts in OSSC values.** Shifts in the values of OSSCs obtained from different methods were quantified using the Welch t-test. The OSSC values obtained from the BDF-3 method and Finite Difference (FD) method were compared against the more accurate ODE15s method (Table S2). The comparison between different methods was done for a z-score of 1 and p-value of 0.01. The largest number of shifts between methods was observed in the Novak and Tyson model.

S-2
