# Supplementary material for: A Test of Highly Optimized Tolerance Reveals Fragile Cell-Cycle Mechanisms Are Molecular Targets in Clinical Cancer Trials
Source: PLoS One. 2008 Apr 23;3(4):e2016. doi: 10.1371/journal.pone.0002016 (PMC2291571; doi:10.1371/journal.pone.0002016)
Supplement: Table S1 — Statistically significant shifts of Overall State Sensitivity Coefficients (OSSCs) between solution methods computed using the Welch t-test. The mean and one standard deviation of the OSSC score computed over the family of random parameter sets is reported. Only shifts recorded with a p-value of 0.01 and z-score of 1 are shown. (0.04 MB DOC) [file pone.0002016.s004.doc]

| **Mechanism** | **ODE15S** | **BDF3** | **FD** |
| --- | --- | --- | --- |
|  |  |  |  |
| **G1/S model** |  |  |  |
| Generation of CycE | 0.6063 ± 0.3502 | 0.4583 ± 0.3364 | 0.5131 ± 0.3476 |
|  |  |  |  |
| **G2/M-DNA Damage model** |  |  |  |
| Generation of p21 | 0.2846 ± 0.1514 | **--** | 0.5016 ± 0.1954 |
| Degradation of p21 | 0.2823 ± 0.1478 | **--** | 0.4847 ± 0.1835 |
|  |  |  |  |
| **Whole cell-cycle model** |  |  |  |
| CycE dependent CycE:Kip1 dissociation | 0.1989 ± 0.2545 | 0.0463 ± 0.0798 | 0.0463 ± 0.0798 |
| CycE:Kip1 dissociation giving Kip1 | 0.1988 ± 0.2545 | 0.0463 ± 0.0798 | 0.0463 ± 0.0798 |
| CycE dependent Kip1 accumulation | 0.1861 ± 0.2386 | 0.0438 ± 0.0744 | 0.0078 ± 0.0371 |
| Degradation of DRGs | 0.1461 ± 0.1720 | 0.0463 ± 0.1020 | **--** |
| Synthesis of p27^Kip1^ | 0.1274 ± 0.1232 | 0.0272 ± 0.0447 | **--** |
| Total E2F concentration | 0.1524 ± 0.1424 | **--** | 0.4249 ± 0.2385 |
| Translational efficiency | 0.6657 ± 0.3816 | **--** | 0.8647 ± 0.2372 |
